# Hepatitis B seroprevalence in 10-25-year-olds in Mexico - the 2012 national health and nutrition survey (ENSANUT) results

**DOI:** 10.1080/21645515.2018.1533617

**Published:** 2018-11-05

**Authors:** Hugo López-Gatell, Lourdes García-García, Gabriela Echániz-Avilés, Pablo Cruz-Hervert, María Olamendi-Portugal, Deyanira Castañeda-Desales, Miguel Ángel Sanchez-Alemán, Martin Romero-Martínez, Rodrigo DeAntonio, Maria Yolanda Cervantes-Apolinar, Ricardo Cortes-Alcalá, Celia Alpuche-Aranda

**Affiliations:** a Centro de Investigación Sobre Enfermedades Infecciosas, Instituto Nacional de Salud Pública, Morelos, México; b Centro de Investigación sobre Evaluación y Encuestas, Instituto Nacional de Salud Pública, Morelos, México; c GSK, Panama City, Panama; d GSK, Mexico City, Mexico

**Keywords:** Hepatitis B, seroprevalence, national health surveys, Mexico, adolescents

## Abstract

**Objectives**: To estimate hepatitis B virus (HBV) seroprevalence from natural infection or vaccination in 10–25-year-olds in Mexico, using the 2012 National Health and Nutrition Survey (ENSANUT).

**Methods**: Randomly selected serum samples (1,581) from adolescents and young adults, representative of 38,924,584 Mexicans, were analyzed to detect hepatitis B surface antigen (HBsAg), hepatitis B surface antibody (anti-HBs) and hepatitis B core antibody (anti-HBc). Weighted HBV seroprevalence in the Mexican population and association with sociodemographic variables were calculated.

**Results**: Overall weighted seroprevalence from natural infection (positive for anti-HBs and anti-HBc) was 0.23% (95% confidence interval [95% CI] 0.10–0.52). No HBsAg was detected, indicating no acute or chronic infection. Vaccine-derived immunity (positive ≥ 10.0 mIU/ml for anti-HBs and negative to anti-HBc) was 44.7% (95% CI: 40.2–49.4) overall; lower in persons aged 20–25 years (40.83%) than in persons aged 10–19 years (47.7%). Among the population analyzed, 54.2% (95% CI: 49.6–58.8) were seronegative to HBV (negative for all three markers) and no sociodemographic risk factors were identified.

**Conclusions**: HBV seroprevalence from natural infection was low. Vaccination-induced immunity was higher among Mexican adolescents than young adults, possibly due to vaccination policies since 1999.

## Introduction

Hepatitis B disease is caused by the hepatitis B virus (HBV), an enveloped virus with a partially double-stranded DNA genome belonging to the family *Hepadnaviridae*. Infection occurs through contact with blood or other body fluids from an infected person. Three major viral antigens are involved in infection and disease propagation: the surface antigen (HBsAg), a glycoprotein forming the virus envelope; the core antigen (HBcAg) which forms the viral capsid; and the envelope antigen (HBeAg) which is mainly secreted into the extracellular space during infection.^1^

HBV infection results in a range of liver diseases, from subclinical infection to severe fulminant hepatitis.^2^ When infection is persistent, chronic disease develops and typically includes liver cirrhosis, hepatocellular carcinoma and its consequences. By detecting viral antigens and antibodies to the viral antigens, it is possible to determine whether a person is chronically infected (with active virus replication), has immunity from natural infection, or has immunity from vaccination.^3^ This information is particularly important in population studies to understand the distribution of HBV in the community, and to identify risk groups that can benefit from prevention and control measures.

The World Health Organization (WHO) estimated that there were 257 million people with chronic HBV infection (HBsAg positive for at least six months) in 2015 with more than 887,000 deaths resulting from hepatitis B (including cirrhosis and liver cancer) globally.^4,5^ Several reports have described HBV seroprevalence in specific groups in Mexico using different methodologies.^6–9^ The only HBV seroprevalence report obtained systematically in a national sample was the National Health Survey (ENSA) in 2000 which included data from adults over the age of 20 years. The seroprevalence of recent HBV infection (HBcAg IgM) was 3.3% (95% CI: 2.8–3.9), and the seroprevalence of chronic infection (presence of HBsAg) was 0.21% (95% CI: 0.11–0.37).^10^

Mexico implemented universal hepatitis B vaccination in 1999. Initially, all infants aged 2, 4 and 6 months received a pentavalent vaccine (i.e., hepatitis B, diphtheria, pertussis (whole cell), tetanus, and *H. influenzae* type b antigens). Starting in 2001, children and adolescents aged 10 to 19 years who were not previously immunized, are eligible for a catch-up hepatitis B vaccine. Since 2007, the hepatitis B vaccine is given as an individual antigen at birth, 2 and 6 months, and was also recommended for use in adolescents and adults at high risk of infection, disease or complications.^11^ Vaccination coverage, estimated from administrative data, for three doses of hepatitis B vaccine in Mexican infants under one year of age ranged from 71% (in 2008) to 99% (in 2012), with an average of 92% in the last 15 years.^12^

The aim of this study was to estimate the seroprevalence of HBV due to natural infection or vaccination-induced immunity using three immunological markers. Data from the National Health and Nutrition Survey (Encuesta Nacional de Salud y Nutrición, ENSANUT) in 2012 were used as a nationally representative sample of adolescents (10 to 19 years old) and young adults (20 to 25 years old).

## Results

Serum samples from 1,581 subjects aged 10 to 25 years old were analyzed, corresponding to a total population of 38,924,584 individuals. There were 1,102 samples representing 22,082,650 individuals aged 10–19 years old, and, 479 samples representing 16,841,935 individuals aged 20–25 years old.

### Acute and chronic infection

No samples were jointly positive for both HBsAg and anti-HBc, considered an index of HBV infection, therefore it was not necessary to differentiate between acute and chronic infection by assessing IgM class antibodies against HBV core antigen (anti-HBc Ag IgM).

### Seropositivity to HBV

#### Immunity attributable to natural infection

Nine individuals, representing 90,254 people in the weighted population, were seropositive for anti-HBs and anti-HBc, but negative for HBsAg, fulfilling the classification criteria for immunity due to natural infection. The corresponding weighted seroprevalence was 0.23% (95% CI: 0.10–0.52) in the overall target population; with 0.3% (95% CI: 0.11–0.79) in adolescents and 0.14% (95% CI: 0.03–0.66) in young adults (Table 1).

**Table 1.** Weighted population and prevalence of biomarkers of hepatitis B serostatus (Mexico, 2012) in 1,581 adolescents (10 to 19 years) and young adults (20 to 25 years) in Mexico, ENSANUT 2012.

		Age group		
		Overall (10–25 years)	10–19 years	20–25 years
Population (N)	Study sample	1,581	1,102	479
	^a^ Weighted population	38,924,584	22,082,650	16,841,935
Seronegative (Negative to all markers)*	Study sample	820	558	262
	^a^ Weighted population	21,107,234	11,303,054	9,804,180
	^a^ % (95% CI)	54.2 (49.6–58.8)	51.2 (45.7–56.7)	58.2 (50.4–65.6)
Immunity from natural infection (positive to anti-HBc and anti-HBs, negative to HBsAg)	Study sample	9	6	3
	^a^ Weighted population	90,254	66,016	24,237
	^a^ % (95% CI)	0.23 (0.10–0.52)	0.30 (0.11–0.79)	0.14 (0.03–0.66)
Immunity from vaccination (positive to anti-HBs, negative to anti-HBs and HBsAg)	Study sample	741	530	211
	^a^ Weighted population	17,413,029	10,535,081	6,877,948
	^a^ % (95% CI)	44.7 (40.2–49.4)	47.7 (42.2–53.3)	40.8 (33.5–48.6)

* HBV serostatus classified according to the US Centers for Disease Control criteria for Interpretation of Serologic Hepatitis B Results

^a^ Estimated numbers and prevalence at national level (STATA SE, v14, for Mac. SVY module); HBV: hepatitis B virus; anti-HBc: hepatitis B core antibody; anti-HBs: hepatitis B surface antibody; HBsAg: hepatitis B surface antigen; 95% CI: 95% confidence interval

#### Immunity attributable to vaccination

A total of 741 individuals, representing 17,413,029 people in the weighted population, were positive for anti-HBs, and negative for anti-HBs and HBsAg, satisfying the classification criteria for immunity due to HBV vaccination. The overall weighted seroprevalence attributable to vaccination was 44.7% (95% CI: 40.2–49.4); with 47.7% (95% CI: 42.2–53.3) in adolescents, and 40.8% (95% CI: 33.5–48.6) in young adults (Table 1).

### Factors associated with HBV serostatus

Women’s sociodemographic characteristics were not significally associated with HBV seroprevalence in the study population.

HBV seroprevalence, overall or due to natural infection or vaccination, was not significantly different in households with low socioeconomic status (SES), medium SES or high SES (p = 0.58). Similarly, there were no significant differences between adolescents and young adults, between men or women, between those living in the North, Center, South or Mexico City, or, between those living in urban versus rural locations (p = 0.1707) for either susceptibility to HBV or immunity from HBV (Table 2). The numbers of subjects who only spoke an indigenous language or who never went to school were too small for a meaningful analysis.

**Table 2.** Hepatitis B serostatus in adolescents (10 to 19 years) and young adults (20 to 25 years) in ENSANUT 2012, by selected sociodemographic characteristics.

	Low SES			Medium SES			High SES			
	n	N	%(N)	n	N	%(N)	n	N	%(N)	p
Seronegative N = 21,107,234	314	6,793,610	52.4	286	6,815,226	52.0	220	7,498,398	58.4	0.58
Immune (natural infection) N = 90,254	5	40,770	0.31	1	24,427	0.19	3	25,057	0.19	
Immune (vaccination) N = 17,413,029	312	6,083,716	47.0	245	6,103,531	46.5	184	5,225,782	40.7	

	Adolescents (10–19 years)			Young adults (20–25 years)			
	n	N	%(N)	n	N	%(N)	p
Seronegative	558	11,303,054	51.2	262	9,804,180	58.2	0.31
Immune (natural infection)	6	66,016	0.3	3	24,237	014	
Immune (vaccination)	530	10,535,081	47.7	211	6,877,948	40.8	
	**Male**			**Female**			**p**
Seronegative	380	11,578,272	59.5	440	9,528,962	49.0	0.0078
Immune (natural infection)	3	43,171	0.22	6	47,083	0.24	
Immune (vaccination)	314	7,813,383	40.1	427	9,599,646	49.3	
	**Urban**			**Rural**			**p**
Seronegative	519	15,347,955	56.1	301	5,759,280	49.8	0.1707
Immune (natural infection)	5	33,935	0.1	4	56,318	0.5	
Immune (vaccination)	440	11,715,782	42.8	301	5,697,247	49.3	

	North			Center			South			Mexico City			
	n	N	%(N)	n	N	%(N)	n	N	%(N)	n	N	%(N)	p
Seronegative	295	6,556,895	59.94	236	7,571,616	55.4	274	6,177,532	49.9	15	801,192	41.9	0.3148
Immune (natural infection)	2	22,726	0.21	4	49,618	0.36	3	17,910	0.14	0	0	0.00	
Immune (vaccination)	232	4,265,859	39.0	227	6,057,012	44.3	268	5,980,578	48.3	14	1,109,580	58.1	

HBV: hepatitis B virus; SES: socioeconomic status; n: number from sample analysed; N: Population size and prevalence for the weighted population at national level (SVY module of STATA SE, V14 for Mac)

When comparing the susceptible group to the group with immunity due to vaccination, no significant differences were found in terms of factors such as: rural versus urban location (adjusted OR $\left[aOR\right]$ 1.29 (95% CI: 0.85–1.95), p = 0.231), living in Mexico City versus the Center (aOR 1.73 (95% CI: 0.53–5.69), p = 0.90), living in the South versus the Center (aOR 1.16 (95% CI: 0.73–1.84), p = 0.63), living in the North versus the Center (aOR 0.86 (95% CI: 0.54–1.38), p = −0.62), or, in those only speaking an indigenous language versus speaking Spanish (aOR 0.27 (95% CI: 0.27–2.82), p = 0.276) (Table 3).

**Table 3.** Correlates of HBV seropositivity due to either natural infection or vaccination in adolescents (10 to 19 years) and young adults (20 to 25 years), ENSANUT 2012.

	Population	Seronegative to HBV^b^ (N)		^a^Weighted Prevalence		Adjusted odds ratio		
Characteristics	Study sample	^a^Weighted population	Study sample	^a^Weighted population	% (95%CI)	Mean	95%CI	P-value
*Gender*								
Male	701	19,464,598	380	11,578,272	59.5(52.3–66.2)	Reference		
Female	880	19,459,987	440	9,528,962	49.0 (43.3–54.6)	1.45	1.01–2.08	0.093
*Age group*								
20–25 years	479	16,841,935	262	9,804,180	58.2 (50.4–65.6)	Reference		
10–19 years	1,102	22,082,650	558	11,303,054	51.2 (45.7–56.7)	1.50	0.95–2.36	0.214
*Type of location*								
Rural	609	11,563,364	301	5,759,280	49.8	Reference		
Urban	972	27,361,220	519	15,347,955	56.1	1.66	0.87–3.18	0.231
*Region*								
Mexico City	29	1,910,772	15	801,192	41.9 (18.7–69.4)	1.73	0.53–5.69	0.90
North	534	10,953,383	295	6,556,895	59.7 (51.7–67.5)	0.86	0.54–1.38	−0.62
Center	468	13,678,246	236	7,571,616	55.4 (46.7–63.7)	Reference		
South	550	12,382,183	274	6,177,532	49.9 (42.7–57.1)	1.16	0.73–1.83	0.63

^a^ Population size and prevalence for the weighted population at national level (SVY module of STATA V14.2 for Mac); HBV: hepatitis B virus; aOR: adjusted odds ratio; 95%CI: 95% confidence interval. ^b^ Seropositive due to natural infection or vaccination. Model included an interaction term for adolescent and urban

## Discussion

### Seroprevalence and infection

This study found no infections and a low seroprevalence of immunity due to natural hepatitis B infection in adolescents and young adults. This suggest that Mexico continues to be a country with low hepatitis B endemicity. WHO has defined low endemicity as < 2% of the population of the general population in a defined geographical area positive for HBsAg”.^13^ Findings are consistent with other studies reporting low seroprevalence of HBV in Mexico and the Latin American region.^10,14–16^ The National Surveillance System reported that the cumulative incidence of hepatitis B per 100,000 from 2005 to 2010 ranged between 0.59 in 2005 up to 1.04 in 2008 in Mexico.^17^ According to a study in six Latin American countries in 1996–1997, seroprevalence of anti-HBc was 21.4% (95% CI: 17.8–25.0) in the Dominican Republic, 7.9% (95% CI: 7.0–8.7) in Brazil, 3.2% (95% CI: 1.7–4.7) in Venezuela, 2.1% (95% CI: 1.4–2.8) in Argentina, 1.4% (95% CI: 1.1–1.7) in Mexico and 0.6% (95% CI: 0.0–1.3) in Chile.^18^

### Seroprevalence attributable to vaccination

This study found 47.7% of adolescents were protected by vaccination compared with 40.8% of young adults. The estimated coverage of the third dose of hepatitis B vaccine in 2000, 2001 and 2002 was 97% for all three years.^12^ According to ENSANUT 2012, the average reported coverage of hepatitis B vaccine in Mexican infants was over 80%.^19^ As specific information on vaccination coverage was not available for these age groups, it was not possible to correlate that information with the seroprevalence findings. However, results of this study suggest that both age groups included had received the hepatitis B vaccine. A conservative seroprotection threshold (i.e., ≥ 10.0 mIU/ml) was used to detect anti-HBs in this study, while other studies have used a threshold of ≥ 5.0 mIU/ml.^20,21^ Therefore, seroprevalence derived from vaccination may have been underestimated. Nonetheless, healthy subjects who have received three doses of vaccination in infancy are considered to have lifelong protection.^22^

### Factors associated with HBV seroprevalence

This study found no sociodemographic factors (e.g. age group, gender, SES, location) significantly associated with seroprevalence of HBV, contrasting with findings from the previous ENSA (2000) analysis that showed households in the lowest income quintile had a higher prevalence of HBV.^10^ Data from ENSA also identified older people (aged 50–59 years versus < 29 years), those living in the South (versus Center) and laborers (versus business owners) as more likely to be seropositive to HBV.^10^ A systematic literature review conducted in 2009 of Mexican studies found transmission was mainly due to sexual transmission and exposure to contaminated surgical equipment or body fluids. There was heterogeneity in seroprevalence levels, as some at-risk groups had higher seroprevalence (e.g., native Mexican populations, those living in poor rural areas or cities with high migration rates, older people, sex workers and healthcare workers).^14^ Previous studies have also identified increased risk of HBV infection in some indigenous groups,^23^ rural environments,^7,8^ chronic liver disease^14^ and health personnel.^24^ Compared to ENSA 2000 and the studies reviewed in 2009,^14^ this study found no samples positive for HBsAg, which suggests a lower circulation of HBV. The nine subjects seropositive due to natural infection (representing 90,254 individuals) were distributed across both age groups, all regions of the country, and both urban and rural locations. Given the small level of seropositivity due to natural infection, this study is underpowered to estimate associations of seroprevalence to other variables. Also, because ENSANUT is a household-based survey targeting the general population, the study design is not suitable to assess the seroprevalence of hepatitis B in specific high-risk groups.

Mexican regulations for vaccination recommend hepatitis B vaccination in populations at high-risk,^25^ similar to vaccination practices in other countries.^26^ However, the risk of HBV infection, disease, and its complications in such groups in Mexico remains unknown. Similarly, the very low seroprevalence of hepatitis B invites to revise whether universal hepatitis B vaccination at birth is a cost-effective preventive intervention.

At the global level, to combat the hepatitis epidemic, the WHO has published its Global Health Sector Strategy against Viral Hepatitis for 2016–2021.^4^ It recommends broadening the coverage of health services and providing care and treatment for those who can benefit from antiviral treatment, for which it is necessary to expand detection programs. Specifically, in areas with intermediate and low endemicity WHO recommends implementing catch-up strategies focused on adolescent cohorts, who may have been born prior to universal vaccination, and adults in high-risk groups. By vaccinating different age groups, the intention is to develop population-level immunity and eventually prevent transmission of the disease in all age groups. Surveillance of acute hepatitis B and national vigilance, such as the present study, are useful to understand the epidemiological pattern of the disease and to evaluate vaccination programs. These data will make possible the identification of opportunities to implement prevention and health promotion measures.^13^

According to the Mexican guidelines for epidemiological surveillance of viral hepatitis, notification of hepatitis B occurs weekly (ICD B16 key) and surveillance is performed through conventional surveillance, outbreak studies, laboratory-based surveillance and surveys.^27^ Although viral hepatitis A, B and C are reported separately, underreporting is likely to occur because etiological diagnosis is not made in every case. The present study, reporting the seroprevalence of three biomarkers of hepatitis B, has the advantage of allowing estimation of the real magnitude of the problem in Mexico.

The main limitation of this study was its cross-sectional design. Data for children under 10 years of age were lacking, therefore, it was not possible to assess seroprotection attributable to immunization of all the population cohorts that have been candidates for vaccination since 1999 and to use it as an indicator of vaccination coverage in Mexico.

## Conclusions

Seroprevalence due to natural HBV infection in Mexico was very low, even among people aged 20–25 years. The group with the highest seropositivity attributable to vaccination was 10 to 19 years of age, which can be explained by the vaccination policies implemented in Mexico since 1999 that targeted infants and adolescents. The findings of this study will inform the revision of public policies for the prevention and control of hepatitis B.

## Methods

### Study design

Methods and core results of ENSANUT 2012 have been described elsewhere.^28^ ENSANUT 2012 is a nationally representative survey of the Mexican population, with a probabilistic, multistage, stratified cluster sampling design. Data and blood from participants were collected at households between October 2011 and October 2012. ENSANUT’s study sample included 50,528 households and 96,031 participants, of which 21,519 were adolescents and 46,303 were adults over 20 years old.^29^

### Power and sample size

Data and sera from 1,581 survey participants, representative of 38,924,584 persons aged between 10 and 25 years old were randomly selected from ENSANUT 2012. Considering a 95% confidence level and a design effect of 1.7, this sample size allows estimating a seroprevalence due to natural infection of 0.46% with an error of 1%, a seroprevalence due to vaccination of 45.8% with an error of 3%.

### Serological analysis

Serum samples were analyzed to detect the following three biomarkers of hepatitis B: 1) hepatitis B surface antigen (HBsAg), 2) hepatitis B surface antibody (anti-HBs), and 3) hepatitis B core antibody (anti-HBc). The commercial ARCHITECT Anti-HBs kit (ABBOTT Diagnostics Division), which has a sensitivity of 97.5% (95% CI: 96.0–98.6%) and a specificity of 99.7% (95% CI: 99.2–99.9) was used to detect anti-HBs. Samples were considered positive when the result was ≥ 10.0 mIU/ml. The commercial ARCHITECT Anti-HBc II kit (ABBOTT Diagnostics Division), which has a sensitivity of 100.0% (95% CI: 99.1–100.0%) and a specificity of 99.7% (95% CI: 99.5–99.8) when analyzing donor samples in blood banks, was used to detect anti-HBc. Samples were considered reactive (positive) when values of the ratio S/CO were ≥ 1 (where S is the sample value and CO is the internal threshold of each batch expressed in relative light units (RLU)). The commercial ARCHITECT HBsAg Quantitative kit (ABBOTT Diagnostics Division), which has a sensitivity of 99.52% (95% CI: 98.29–99.94%) and a specificity of 99.87% (95% CI: 99.74–99.94%) when analyzing samples from six clinical laboratories, was used to detect HBsAg. Samples were considered reactive (positive) for values ≥ 0.05 IU/ml. Samples were processed according to the manufacturers of diagnostic equipment instructions, at the Center for Infectious Diseases Research of the National Institute of Public Health. Laboratory personnel were blinded to the subjects’ characteristics.

### Definition of variables

HBV serological results were classified according to the Interpretation of Hepatitis B Serologic Tests Results, as published by the US Centers for Disease Control and Prevention (CDC).^30^ Persons defined as seronegative to HBV were negative to all three markers studied (anti-HBs, anti-HBc and HBsAg). Persons defined as immune due to natural infection were positive to anti-HBs and anti-HBc, and negative to HBsAg. Immunity due to vaccination was defined when samples were positive to anti-HBs, and negative to anti-HBc and HBsAg. HBV infection was defined when samples were positive to HBsAg and anti-HBc, and negative to anti-HBs. Reported place of residency was classified into four country regions: North, Center, South and Mexico City (Appendix 1). Locations were divided into urban (≥ 2,500 inhabitants) and rural (< 2,500 inhabitants). A distinction was made between those who ever attended school and those who never attended, and between those who only speak an indigenous language and Spanish speakers.

### Statistical analysis

Descriptive analyses were performed for the overall population (10–25 years), and two age strata: adolescents (10–19 years) and young adults (20–25 years). Multinomial logistic regression models were fitted to estimate the association of gender, age category and sociodemographic characteristics (i.e., geographic region, urban or rural setting, indigenous language spoken, and schooling) with immunity due to natural infection or vaccination, compared to the seronegative group in the overall sample, adjusted for all other variables. Multivariate logistic regression models were also fitted for a binomial indicator variable classifying individuals as positive due to either infection or vaccination vs. negative. Model selection was driven by the overall goodness of fit and the theoretical relevance of the variables. The final model was adjusted by age group, gender, region, type of location and indigenous language. Adjusted odds ratios (aORs) were estimated to compare sociodemographic characteristics of the immune and susceptible groups. The population size, seroprevalence of HBV biomarkers, and aORs were estimated for the weighted population using the STATA survey analysis module (svy), with sampling weights proportional to the probabilities of selection into ENSANUT 2012 and this seroprevalence study. The 95% CI around mean seroprevalence and aORs were regarded as a measure of uncertainty. Data were analyzed in STATA SE for Mac, version 14.2.

### Ethical considerations

The protocol for performing the ENSANUT 2012 and the specific protocol for analysis of sera for this study were approved by the Research, Biosafety and Ethics committees of the National Institute of Public Health. Informed consent was obtained from participants. Data collection and management followed principles of confidentiality, and were consistent with current legislation for the protection of personal data.

## Highlights

- 54.2% of adolescents and young adults were seronegative to HBV infection in Mexico in 2012.
- Seroprevalence from HBV infection was low (0.23%), representing 90,254 Mexicans.
- Vaccine-induced seroprotection was 47.7% among adolescents and 40.8% among young adults.

No evidence of chronic HBV infection in this population aged 10-25 years old.
