# Supplementary material for: Hepatitis B seroprevalence in 10-25-year-olds in Mexico - the 2012 national health and nutrition survey (ENSANUT) results
Source: Hum Vaccin Immunother. 2018 Nov 5;15(2):433–9. doi: 10.1080/21645515.2018.1533617 (PMC6422518; doi:10.1080/21645515.2018.1533617)
Supplement: Supplemental Material [file khvi-15-02-1533617-s001.docx]

# Appendix 1. States in each Mexican region

**North:**Baja California, Southern Baja California, Chihuahua, Coahuila, Durango, Nuevo Leon, Sonora, Sinaloa, Tamaulipas and Zacatecas

**Center:**Aguascalientes, Colima, Guanajuato, Hidalgo, Jalisco, State of Mexico, Michoacán, Nayarit, Querétaro, San Luis Potosí and Tlaxcala

**Mexico City**

**South**: Campeche, Chiapas, Guerrero, Morelos, Oaxaca, Puebla, Quintana Roo, Tabasco, Veracruz and Yucatán
